# Insight into Bioactive Compounds and Antioxidant Activity of Bakery Products Fortified with Fruit Pomace

**DOI:** 10.3390/foods14050806

**Published:** 2025-02-26

**Authors:** Maria Bianca Mandache, Loredana Elena Vijan, Sina Cosmulescu

**Affiliations:** 1 Doctoral School of Plant and Animal Resources Engineering, Faculty of Horticulture, University of Craiova, 13 A.I. Cuza Street, 200585 Craiova, Romania; mandachemaria@yahoo.com; 2 Faculty of Sciences, Physical Education and Computer Science, The National University of Science and Technology Politehnica Bucharest, Pitesti University Centre, 1 Targu din Vale Street, 110040 Pitesti, Romania; 3 Department of Horticulture and Food Science, Faculty of Horticulture, University of Craiova, 13 A.I. Cuza Street, 200585 Craiova, Romania

**Keywords:** apple pomace, sour cherry pomace, peach pomace, phenolic compounds, antiradical activity

## Abstract

The aim of this work was to analyse the influence of the application of apple, sour cherry and peach pomace on the content of bioactive compounds and antioxidant activity in bakery products supplemented with different proportions of pomace (5%, 10% and 15%). The type of pomace applied determined some variations, but it was observed that at a maximum substitution percentage (15%), breads with peach pomace recorded the highest levels of polyphenols (855.10 mg GAE/100 g), flavonoids (181.01 mg CE/100 g) and tannins (385.26 mg GAE/100 g), which also generated the highest antiradical activity (42.84%). In general, an evolutionary distribution was observed between the increase in the content of polyphenols, flavonoids, tannins and antioxidant activity, which could indicate a synergistic action between all components. In all the analysed samples, increases in the constituent compounds and antioxidant activity were observed, consistent with the percentage of added pomace, with the analysis of the inclusion of different types of pomace becoming relevant in the context of industrial production.

## 1. Introduction

White bread is a common part of many people’s diets and is considered a staple food worldwide. It is a significant source of carbohydrates, such as starch, but is low in dietary fibre and essential nutrients [1], with general analyses of nutritional value even indicating a chemical imbalance [2]. Recently, consumers have shown increased interest in healthy food rich in nutrients [3], and with this interest, industrial processors are pursuing, and at the same time have the opportunity to configure, a new food system. At the same time, the UN has established new recommendations for the human population, among the 17 major goals of the 2030 Agenda, including “Responsible Consumption and Production” and “Health and Wellbeing” [4]. Epidemiological studies on human health have emphasised the beneficial role of phenolic compounds in plant materials in preventing a wide range of diseases [5,6,7]. These compounds are found mainly in plant materials. Among these, fruit pomace stands out for its complex content of essential bioactive compounds, such as dietary fibre, polysaccharides, phenolic compounds, phytochemicals and natural antioxidants, which support health by optimising cellular and metabolic activities, due to their anti-inflammatory properties, antioxidant effect, anti-cancer and anti-allergenic potential [8]. Although oxidation processes in the human body are a natural biological reaction, the resulting free radicals can cause damage to cell membranes and other cellular structures [9,10,11]. In the long term, the damage caused by excess free radicals in the body can become irreversible, leading to the development of cardiovascular and liver diseases and even certain types of cancer [2]. Pecyna et al. [12] states that the development of a new type of bread supplemented with biologically active substances, including plant antioxidants, could be a very important nutritional support for human health. Therefore, the aim of this work is to evaluate the influence of integrating apple, sour cherry and peach pomace in the production process of bakery products, in order to improve their functional properties. Thus, the work aims to contribute to the development of healthier and more sustainable bakery products, while responding to the challenges related to waste management in the food industry.

## 2. Materials and Methods

### 2.1. Plant Material

Apple, sour cherry and peach pomace, obtained from the cold pressing process of the fruits, was collected from three fruit and vegetable juice producers. It was then dehydrated in an industrial dehydrator at a temperature between 50 °C and 60 °C until the moisture level became constant, then ground using an electric grinder and stored at a temperature of 4 °C in hermetically sealed glass containers.

### 2.2. Experimental Variants

To make the control bread, the raw and auxiliary materials used included the following: wheat flour type 000 (250 g), Pakmaya yeast for baking (*Saccharomyces cerevisiae*) (4 g), salt iodized sodium chloride) (4 g), and drinking water (150 mL).

In the fortified bread samples, part of the white wheat flour (5%, 10% and 15%) was substituted with apple, sour cherry and peach pomace powder (12.5 g, 25 g and 37.5 g) to obtain the following formulations: Pm (bread with added apple pomace) 5, 10 and 15%; Pv (bread with added sour cherry pomace) 5, 10 and 15%; and Pp (bread with added peach pomace) 5, 10 and 15% (Table 1).

The technological process of obtaining bakery products in the bakery included kneading for 6 min, fermentation for 15 min, dividing and shaping the dough, rising at 35 °C and 75% humidity (60 min for the control bread and 90 min for the bread with pomace), scoring and baking at 250 °C for 25 min in a hearth oven.

### 2.3. Methods of Analysis

#### 2.3.1. Preparation of Extracts Specific for the Quantification of Polyphenols, Flavonoids and Anthocyanins

For the determination of polyphenols, flavonoids and anthocyanins, the bread (1 g) was vortexed with 10 mL of absolute ethanol (Merck-Sigma-Aldrich, Darmstadt, Germany) for 2 min (Vortex Mixer VX-200 Corning-Labnet, Corning Life Sciences, Tewksbury, MA, USA), ultrasonication at 40 kHz for (ULTR-2L0-001, Labbox Labware, Migjorn, Spain) for 30 min and centrifuged at 6000 rpm (Labnet Spectrafuge 6c, Labnet International Inc., Edison, NJ, USA) for 30 min. The process was repeated with a new cycle of vortexing, ultrasonication and centrifugation under the same conditions.

#### 2.3.2. Preparation of Extracts Specific for the Quantification of Tannins and Sugars

For the quantitative determination of tannins and sugars, aqueous solutions of the experimental material were prepared by vortexing 1 g of bread in 10 mL of water for 2 min. The samples were ultrasonicated at 99 °C for 30 min.

#### 2.3.3. Determination of Total Polyphenol Content

The total polyphenol content was determined spectrophotometrically (UV-Vis Perkin Elmer Lambda25, Shelton, CT, USA) according to a method adapted from Cosmulescu et al. [13]. The samples were prepared by mixing 2 mL the ethanol extract of bread with 5.5 mL distilled water, 0.5 mL Folin-Ciocâlteu reagent and 2 mL sodium carbonate solution 10% (Merck-Sigma-Aldrich, Darmstadt, Germany), then left for 2 h in the dark. Absorbance was measured at 765 nm, and the results expressed in mg equivalent to gallic acid equivalent (GAE)/100 g sample, using the gallic acid calibration curve.

#### 2.3.4. Determination of Total Flavonoid Content

The flavonoid content was determined according to a method adapted from Stamin et al. [14]. The ethanolic extract of bread 2 (mL) was mixed with 5 mL of distilled water, 0.5 mL of 5% sodium nitrite, 0.5 mL of 10% aluminium chloride and 0.5 mL of 1 M sodium hydroxide (Merck-Sigma-Aldrich, Darmstadt, Germany). After shaking, the absorbance was measured at 510 nm, and the flavonoid concentration expressed in mg equivalent to catechin equivalent (CE)/100 g sample, using the catechin calibration curve.

#### 2.3.5. Determination of Total Anthocyanin Content

The anthocyanin content was determined according to the methodology adapted from Stamin et al. [14]. The analysis was performed using 1 mL ethanolic extract of bread, 1 mL of absolute ethyl alcohol solution acidified with 0.1% pure HCl and 8 mL of pH 0.6 buffer solution (2% HCl solution) or pH 3.5 buffer solution (21.7 g of disodium phosphate and 14.6 g of citric acid dissolved in distilled water). Absorbance was measured at 520 nm, and the results were expressed as mg equivalent to cyanidin 3-glucoside equivalent (C3G)/100 g sample, using the C3G calibration curve.

#### 2.3.6. Determination of Total Tannin Content

The concentration of tannins was determined using the method described by Stamin et al. [14]. Samples were prepared by mixing 2 mL of aqueous extract with 5.5 mL of distilled water, 0.5 mL of Folin–Ciocâlteu reagent and 2 mL of 10% sodium carbonate solution (Merck-Sigma-Aldrich, Darmstadt, Germany). After stirring and standing for one hour in the dark, the absorbance was measured at 765 nm. The total tannin content was expressed as mg equivalent to gallic acid equivalent (GAE)/100 g sample, using the gallic acid calibration curve.

#### 2.3.7. Determination of Total Sugar Content

The sugar content was determined spectrophotometrically according to the phenol-sulphuric acid method [14], with slight changes. The aqueous extract (0.2 mL) was mixed with 0.8 mL of distilled water, 5 mL of concentrated sulfuric acid, 1 mL of aqueous solution with 0.5 mL of phenol 5% (Merck-Sigma-Aldrich, Darmstadt, Germany). Afterwards, it was shaken vigorously for colour development. The absorbance was measured at 490 nm and the total sugar content was expressed as mg glucose equivalent (GluE)/100 g sample, using the glucose calibration curve.

#### 2.3.8. Determination of Components with Antiradical Potential

The radical scavenging activity (RSA%) of the ethanolic extracts was determined using the DPPH assay, which is based on the transfer of hydrogen from the antioxidant to the DPPH radical, causing a change in the colour of the solution from purple to yellow and a reduction in absorbance at 517 nm. The assay involved mixing 3 mL of DPPH solution in methanol (Merck-Sigma-Aldrich, Darmstadt, Germany) with 0.1 mL of ethanolic extract, followed by shaking and keeping at ambient temperature for 20 min in the dark. Absorbance was measured at 517 nm, and the percentage of DPPH radical scavenging activity (RSA%) was calculated using the formula:

y = [1 − (sample absorbance/control absorbance)] × 100, where the control absorbance is the absorbance of the stock solution of DPPH at 517 nm [15,16].

### 2.4. Statistical Analysis

All determinations were performed in triplicate (n = 3), and the identified results were expressed as mean ± standard deviation (X ± SD), using Microsoft Excel 2010 software. Statistical analysis was performed using the IBM SPSS Statistics 26 software, including one-way and two-way ANOVA and Duncan’s multiple range tests (*p* < 0.05).

The analysis of the relationship between the biochemical compounds identified in the bread varieties and RSA% was performed using a “radar chart”. Each axis represents a bread variety (Pm, Pv and Pp), and the absolute values were converted into relative units reported to the concentration of the control sample. RSA values were also expressed relative to the control sample, with all values on the vertical axis equal to 1.

## 3. Results and Discussion

The results obtained in this study highlight the impact of the integration of apple, sour cherry and peach pomace on the content of bioactive compounds (polyphenols, flavonoids, anthocyanins, tannins, sugars) and antioxidant activity (RSA%) in bread with different concentrations of pomace, providing a detailed perspective on the functional benefits and added value of this innovative ingredient.

Table 2 presents the results regarding the content of polyphenols, flavonoids, tannins, sugars and antioxidant activity of the formulated breads. Although decreases that may occur following thermal processing were anticipated [17], supplementation with apple, cherry and peach pomace resulted in a significant upward expansion of the polyphenol concentration. Predictably, the lowest level was evident in bread without addition (349.54 mg GAE/100 g). Formulations with a minimum level of pomace (5%), recorded the lowest values of phenolic compounds (479.42, 482.86 and, respectively, 549.99 mg GAE/100 g), regardless of the type of pomace used, compared to those that integrated maximum concentrations of pomace (15%), where the values obtained varied between 629.78 mg GAE/100 g and 855.10 mg GAE/100 g.

Thus, this difference between the values of phenolic compounds at different concentrations of pomace directly reflects the proportionality between the amount of pomace added and the content of phenolic compounds in bakery products. Naturally, a higher amount of pomace will add a higher amount of phenolic compounds and antioxidants. Increasing the concentration of pomace in the formula allows for a more efficient integration of these compounds into the final product, which explains the higher values observed at concentrations of 15%.

Compared to the values reported by Gumul et al. [18], this study revealed higher concentrations of phenolic compounds in apple pomace bread. Previous studies have shown that as the concentration of apple pomace increases (5–15%), the polyphenol content of gluten-free bread increases significantly [19]. Similar results were obtained by Valková et al. [20] and Gumul et al. [21], confirming the positive effect of pomace on the concentration of bioactive compounds, including polyphenols.

Regarding the type of pomace, in our study peach pomace (Pp) consistently presented the highest values, regardless of the concentration, followed by sour cherry (Pv) and apple (Pm). This may be due to the chemical composition of the pomace, or the typology of the phenolic compounds. Different types of pomace may contain different types of phenolic compounds, which may vary depending on their ability to be preserved during the drying or processing process [22].

**Figure 1:**
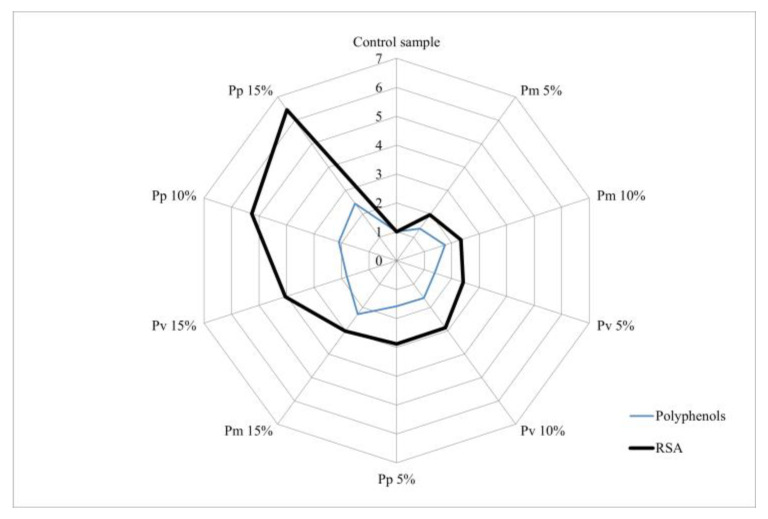
Radar diagram of the influence of polyphenol content on antioxidant activity.

According to the radar diagram representation (Figure 1), a progressive distribution of the level of polyphenols and antioxidant activity can be observed in relation to the concentration of pomace applied (0, 5, 10, 15%), but the lack of a proportional relationship of their growth level is also highlighted, suggesting that polyphenols are not the only factors involved in the antioxidant capacity values.

The inclusion of different concentrations of fruit pomace in the recipe for the manufacture of bakery products also produced a significant increase in the total flavonoid content (Table 2). Compared to the control sample (49.87 mg CE/100 g), an increase in the total flavonoid content was observed depending on the type and amount of pomace added, the range of variations including minimum values at 5% pomace (86.68, 82.99 and 136.83 mg CE/100 g) and maximum values at 15% pomace (139.61, 120.45 and 181.01 mg CE/100 g). Similarly, in the study conducted by Gumul et al. [19], an increase in flavonoid content in supplemented bread samples is illustrated, determined by the percentage of apple pomace applied, with values ranging between 8.04 mg rutin/100 g (5%) and 21.56 mg rutin/100 g (15%). Analogous to previous findings, it is observed that with the increase in pomace concentrations (from 5% to 15%), the total flavonoid content gradually increases, bringing a positive impact on the finished products.

**Figure 2:**
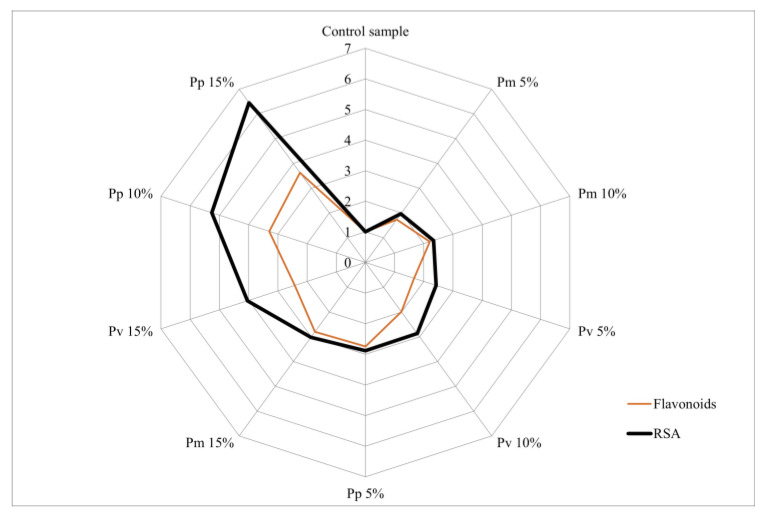
Radar diagram of the influence of flavonoid content on antioxidant activity.

According to the radar diagram analysis (Figure 2), it can be noted the existence of a direct relationship between the total flavonoid content and the antioxidant activity (RSA%), with the highest values recorded at the maximum pomace concentration (15%). This influence was more pronounced in the case of peach pomace (Pp), which also presented the highest flavonoid concentrations, followed by sour cherry pomace (Pv) and, respectively, apple pomace (Pm).

The amount of added pomace also influenced the anthocyanin concentration (Table 2), which, in general, varied significantly. Compared to the control sample (6.15 mg C3G/100 g), a progressive but statistically insignificant increase in the total anthocyanin content was observed in breads with apple pomace between the samples with 10% and 15% addition. The developments identified in breads with apple pomace were determined by the increase in the weight of powder included (5–15%) and included values that fluctuated between 15.26 and 16.12 mg C3G/100 g. The results of the present study are superior to those reported by Gumul et al. [21], who identified in bakery products with apple pomace a level of anthocyanins between 0.10 mg C3G/g (control sample) and 0.143 C3G/g (15%).

The same upward trend is also observed in breads with sour cherry and peach pomace, where values ranging from 12.48 to 14.95 mg C3G/100g (5–15%) and 6.59–8.92 mg C3G/100 g (5–15%), respectively, were detected. In relation to the type of pomace applied, it was found that, at each level of substitution, breads with apple pomace had the highest anthocyanin content, followed by those with sour cherry and peach pomace, the variations observed clearly indicating the effect of the inclusion of pomace in the chemical composition of the finished products.

**Figure 3:**
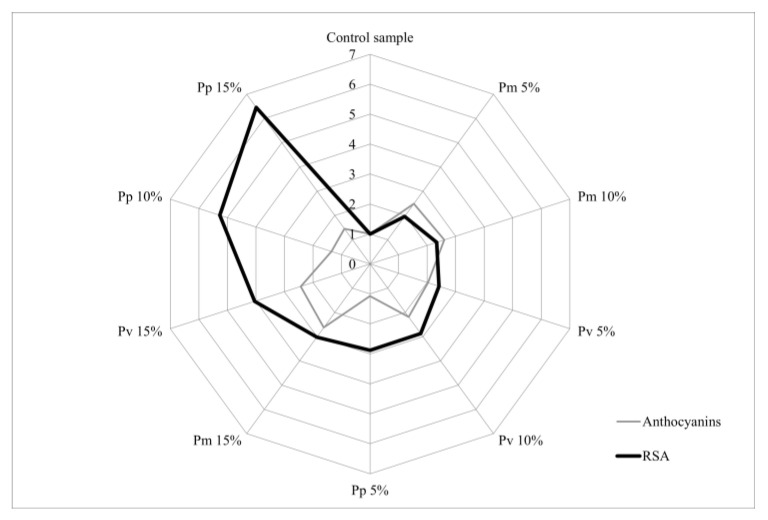
Radar diagram of the influence of anthocyanins content on antioxidant activity.

According to the diagram shown in Figure 3, it can be observed that the levels of anthocyanins did not show the same linearity, indicating the existence of a disproportionality with respect to the antioxidant capacity. These variations can be explained by the particularities of the distinctive phenolic composition of each type of pomace analysed. In general, fruits are considered major sources of anthocyanins and their consumption also implies the intake of certain concentrations of antioxidants [23]. However, their diversity in phytochemical compounds and vitamins can cause interactions with anthocyanins in a synergistic or antagonistic way, which can produce an increase or decrease in antioxidant activity [24].

In accordance with the anticipated trends, the control sample (57.44 mg GAE/100 g) recorded the lowest concentrations of tannins (Table 3), followed by the bread formulations in which a quantity of 5% pomace was integrated (103.96, 110.11 and, respectively, 157.84 mg GAE/100 g). The addition of 15% pomace resulted in a substantial increase in the total tannin content of the supplemented breads, with values ranging from 147.77 to 385.26 mg GAE/100 g. Among all the analysed samples, peach pomace was distinguished by significantly higher concentrations of tannins, compared to sour cherry pomace, but also to apple pomace, which presented the lowest content. Thus, the fluctuations identified between the different types of pomace highlight the variety of constituent compounds of these by-products. The highest tannin values were recorded at a 15% increase in the proportion of pomace, which may highlight a direct relationship between increasing concentration and improving the nutritional value of the final product.

**Figure 4:**
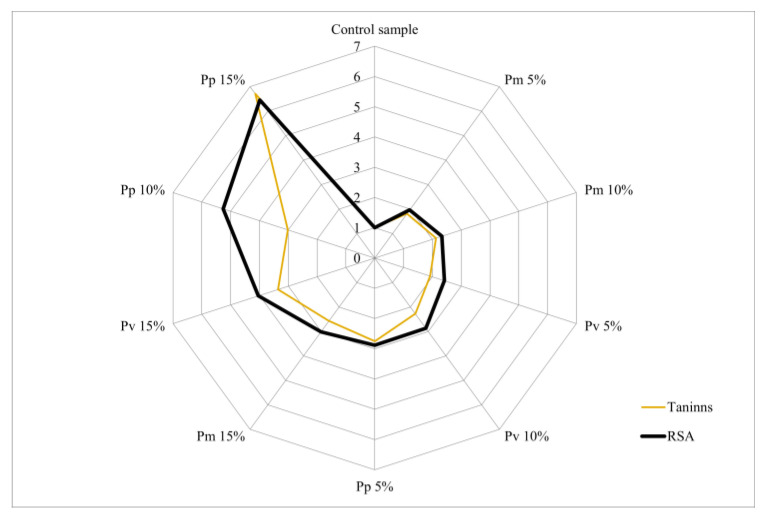
Radar diagram of the influence of tannins content on antioxidant activity.

Radar diagram analysis (Figure 4) highlighted the positive influence of increasing tannin concentrations on antioxidant capacity. Overall, a uniform progressive distribution of tannin levels and antioxidant activity was observed in relation to the increasing percentage of integrated pomace (0, 5, 10, 15%), but also a variability determined by the type of pomace.

The type and amount of integrated pomace conditioned the sugar content (Table 3), which fluctuated in all the analysed samples. Their level increased gradually, in the breads with the addition of 5 and 15% pomace, with concentrations between 27.48 and 28.67 g GluE/100 g (Pm); 19.06–23.18 g GluE/100 g (Pv); and 10.42–11.27 g GluE/100 g (Pp). The concentration of sugars detected in the breads with 5% and 10% apple pomace did not differ considerably, but compared to the control sample (7.28 g GluE/100 g), significant increases were found. The highest values were detected in apple pomace, followed by sour cherry and peach pomace, with the oscillations identified between the contents being attributed to the distinct chemical composition of the pomace which has a different level of sugars. Also, during the fermentation process, yeast activity is influenced by the presence of higher concentrations of carbohydrates, leading to a more intense conversion of starch into simple sugars [25]. Thus, this process is clearly noticeable in the case of 15% addition, where the concentrations reach maximum values.

Gumul et al. [21] states that fruit pomace is a natural concentrate, abundant in endogenous antioxidants, capable of providing antioxidant properties to fortified products. According to the data presented (Table 3), the control sample (6.64%) presented the lowest antiradical activity, compared to the supplemented variants. The addition of pomace generated significant increases, dependent on the applied concentration, in the breads with peach pomace, the highest antiradical activity being outlined, with a degree of inhibition ranging between 19.13% (5%) and 42.82% (15%). Similarly, the breads enriched with apple and sour cherry pomace showed the same growth trend, with inhibition ranges that ranged between 13.03 and 20.04% (5–15%) and, respectively, 16.05–26.83% (5–15%). The high concentrations of phenolic compounds in the used pomace were reflected in the level of antioxidants found in the bakery products. Compared to the control sample, the formulations varied significantly, but some similarities were also found between the breads with 5% peach pomace, 10% sour cherry and 15% apple pomace.

Of all the samples analysed, peach pomace showed higher antiradical activity compared to apple and sour cherry pomace, highlighting its superior capacity to neutralise free radicals and protect the human body against oxidative stress. Thus, we can highlight that the integration of fruit pomace into the formulation of bakery products directly contributes to the increase in antioxidant activity of the final products, a finding that is consistent with the studies conducted by Mir et al. [26] and Cantero et al. [27]. Similar results were reported by Valková et al. [20] and Gumul et al. [19], who observed that the antioxidant activity of breads supplemented with apple pomace increased from 1.65 g Trolox/kg (control bread) to 2.79 g Trolox/kg (10%) and from 0.03 mg Trolox/g (control bread) to 3.21 mg Trolox/g (15%), respectively.

Fruit pomace can be a valuable ingredient for the food industry due to its high concentration of phytochemical compounds and high antioxidant capacity, as outlined by Zaky et al. [28], and with its integration into the food manufacturing process, new innovative food products could be developed.

## 4. Conclusions

In conclusion, the results of this research attest to the possibility of using apple, sour cherry and peach pomace as a direct source of biochemical substances, which can be integrated into food products. The detailed analysis of the chemical composition, as well as the optimisation of the experimental variants, highlighted its potential for use in the food processing industry. The integration of variable concentrations (5, 10 and 15%) of fruit pomace into the manufacturing recipe of bakery products had the effect of a directly proportional increase in bioactive compounds and antioxidant activity in most of the finished products. The type of pomace significantly influenced the chemical composition of the products, with breads with peach pomace having the highest antioxidant activity and the highest concentrations of polyphenols, flavonoids and tannins. These ingredients, generated by the juice processing industry, offer promising opportunities for improving the nutritional profile of foods, supporting current trends in sustainable and functional nutrition.

## Figures and Tables

**Table 1:** Raw and auxiliary materials used in bread formulation and experimental variants.

Raw and Auxiliary Materials	Control Bread	Bread with Pomace (Pm, Pv, Pp) 5%	Bread with Pomace (Pm, Pv, Pp) 10%	Bread with Pomace (Pm, Pv, Pp) 15%
Wheat flour	250 g	237.5 g	225 g	212.5 g
Apple pomace powder	0	12.5 g	25 g	37.5 g
Sour cherry pomace powder	0	12.5 g	25 g	37.5 g
Peach pomace powder	0	12.5	25 g	37.5 g
Yeast	4 g	4 g	4 g	4 g
Water	150 mL	150 mL	150 mL	150 mL
Salt	4 g	4 g	4 g	4 g

Pm: bread with added apple pomace; Pv: bread with added sour cherry pomace; Pp: bread with added peach pomace.

**Table 2:** The composition of polyphenols, flavonoids and anthocyanins of wheat bread with a share of apple, sour cherry and peach pomace.

Sample	Concentration	Polyphenols (mg GAE/100 g)	Flavonoids (mg CE/100 g)	Anthocyanins (mg C3G/100 g)
Control sample	0%	349.54 ± 0.77 ^j^	49.87 ± 1.27 ^j^	6.15 ± 0.08 ^i^
Bread with added apple pomace (Pm)	5%	479.42 ± 0.73 ^i^	86.68 ± 1.30 ^h^	15.26 ± 0.06 ^b^
	10%	613.15 ± 1.43 ^e^	110.09 ± 1.48 ^f^	16.03 ± 0.06 ^a^
	15%	799.53 ± 2.91 ^b^	139.61 ± 1.25 ^c^	16.12 ± 0.03 ^a^
Bread with added sour cherry pomace (Pv)	5%	482.86 ± 0.43 ^h^	82.99 ± 1.21 ^i^	12.48 ± 0.09 ^e^
	10%	558.26 ± 0.86 ^f^	99.28 ± 1.50 ^g^	13.46 ± 0.07 ^d^
	15%	629.78 ± 1.01 ^d^	120.45 ± 1.72 ^e^	14.95 ± 0.06 ^c^
Bread with added peach pomace (Pp)	5%	549.99 ± 0.54 ^g^	136.83 ± 1.94 ^d^	6.59 ± 0.06 ^h^
	10%	732.91 ± 2.43 ^c^	164.39 ± 2.07 ^b^	8.31 ± 0.08 ^g^
	15%	855.10 ± 3.04 ^a^	181.01 ± 0.54 ^a^	8.92 ± 0.08 ^f^

Distinct letters indicate statistically significant differences (Duncan test with multiple intervals, *p* < 0.05).

**Table 3:** The composition of tannins, sugars and antioxidant activity of wheat bread with a share of apple, sour cherry and peach pomace.

Sample	Concentration	Tannins (mg GAE/100 g)	Sugars (g GluE/100 g)	RSA (%)
Control sample	0%	57.44 ± 1.14 ^j^	7.28 ± 0.12 ^h^	6.64 ± 0.55 ^g^
Bread with added apple pomace (Pm)	5%	103.96 ± 1.35 ^i^	27.48 ± 0.30 ^b^	13.03 ± 0.91 ^f^
	10%	122.10 ± 1.43 ^g^	27.82 ± 0.29 ^b^	15.49 ± 0.56 ^e^
	15%	147.77 ± 1.76 ^e^	28.67 ± 0.41 ^a^	20.04 ± 0.70 ^d^
Bread with added sour cherry pomace (Pv)	5%	110.11 ± 0.31 ^h^	19.06 ± 0.14 ^e^	16.05 ± 0.48 ^e^
	10%	130.83 ± 1.11 ^f^	22.09 ± 0.17 ^d^	19.04 ± 0.55 ^d^
	15%	192.82 ± 2.19 ^b^	23.18 ± 0.25 ^c^	26.83 ± 0.89 ^c^
Bread with added peach pomace (Pp)	5%	157.84 ± 1.03 ^d^	10.42 ± 0.12 ^g^	19.13 ± 0.66 ^d^
	10%	173.04 ± 0.80 ^c^	10.69 ± 0.24 ^g^	34.95 ± 0.57 ^b^
	15%	385.26 ± 1.75 ^a^	11.27 ± 0.39 ^f^	42.84 ± 0.56 ^a^

Distinct letters indicate statistically significant differences (Duncan test with multiple intervals, *p* < 0.05).

## Data Availability

The original contributions presented in this study are included in the article. Further inquiries can be directed to the corresponding authors.

## Abbreviations

PM	Bread with added apple pomace.
PV	Bread with added sour cherry pomace.
PP	Bread with added peach pomace.
